# Prognostic Impact of Histologic Subtypes in Mismatch Repair‐Deficient/Microsatellite Instability‐High Colorectal Cancer: A Single‐Center Retrospective Study of 1127 Stage 0–IV Patients

**DOI:** 10.1002/ags3.70159

**Published:** 2025-12-30

**Authors:** Tomoyuki Momma, Hirokazu Okayama, Sohei Hayashishita, Hiroya Suzuki, Masanori Katagata, Takuro Matsumoto, Daisuke Ujiie, Shun Chida, Wataru Sakamoto, Koji Kono

**Affiliations:** ^1^ Department of Gastrointestinal Tract Surgery Fukushima Medical University School of Medicine Fukushima Japan; ^2^ Department of Gastrointestinal Surgery, Aizu Medical Center Fukushima Medical University Fukushima Japan

**Keywords:** colorectal cancer, deficient mismatch repair/microsatellite instability‐high (dMMR/MSI‐H), histological type, relapse‐free survival

## Abstract

**Background:**

Deficient mismatch repair/microsatellite instability‐High (dMMR/MSI‐H) colorectal cancer (CRC) generally confers a favorable survival yet is paradoxically enriched for poorly differentiated (Por) histology, which is linked to aggressive behavior. We evaluated how MMR/MSI status and histological subtype relate to stage distribution and prognosis.

**Methods:**

We conducted a single‐center retrospective study of 1127 Stage 0–IV CRCs. MMR/MSI status was assessed by immunohistochemistry (MMR‐IHC) and/or MSI testing. Clinicopathological features and relapse‐free survival (RFS) were analyzed, with survival analyses focused on 900 curatively resected Stages I–III cases.

**Results:**

MMR‐IHC and MSI testing showed 98.4% concordance; 104 patients (9.2%) had dMMR/MSI‐H tumors. In proficient MMR/microsatellite stable (pMMR/MSS) CRC, well/moderately differentiated (Wel/Mod) histology predominated (~90%) across all stages. By contrast, dMMR/MSI‐H tumors exhibited a stage‐dependent histological shift, with Por histology increasing from 11.5% in Stage I to 60.0% in Stage IV. Among Stage I–III patients, dMMR/MSI‐H was associated with superior RFS compared with pMMR/MSS CRCs, with statistical significance confined to Stage II. In Stage III, Por histology was strongly associated with early relapse and remained an independent adverse factor in multivariable models. Notably, within Stage III dMMR/MSI‐H CRC, Por histology was associated with distinctly worse RFS, whereas no recurrences were observed among Wel/Mod cases.

**Conclusion:**

While confirming established prognostic trends, this study highlights that dMMR/MSI‐H CRC is histologically heterogeneous. A Por subset, prevalent in advanced disease, appeared to attenuate the survival advantage and was associated with early recurrence in Stage III, although validation is required due to limited sample sizes in subgroups.

## 1 Introduction

Despite remarkable advancements in endoscopic therapy, surgical techniques, systemic chemotherapy, and the revolutionary introduction of immunotherapy, colorectal cancer (CRC) remains the second leading cause of cancer‐related mortality worldwide [1], underscoring the critical need for further elucidating its molecular and clinicopathological underpinnings to optimize patient management. Mismatch repair‐deficient (dMMR) or microsatellite instability‐high (MSI‐H) CRC represents a distinct molecular subgroup, accounting for approximately 15% of all CRCs. These tumors arise either from germline mutations in MMR genes (Lynch syndrome), or more commonly, from sporadic mechanisms, notably epigenetic silencing via somatic hypermethylation of the MLH1 gene promoter. Compared with microsatellite stable (MSS) or mismatch repair‐proficient (pMMR) CRCs, dMMR/MSI‐H tumors exhibit a unique clinicopathological profile, characterized by a predominance in the proximal (right‐sided) colon, a higher frequency of mucinous (Muc) or poorly differentiated (Por) histology, and a prominent infiltration of tumor‐infiltrating lymphocytes (TILs) [2, 3]. Furthermore, sporadic dMMR/MSI‐H CRCs frequently harbor the BRAF V600E mutation.

A key characteristic of dMMR/MSI‐H CRC is its varying prevalence across disease stages: it is typically detected in approximately 20% of Stage II tumors, decreases to about 10% in Stage III, and is found in only 4%–5% of metastatic (Stage IV) cases [2, 4, 5]. Importantly, the prognostic impact of dMMR/MSI‐H status in CRC is highly stage‐dependent. In postoperative Stage II disease, extensive evidence consistently indicates that dMMR/MSI‐H CRCs confer a more favorable prognosis than pMMR/MSS CRCs [5, 6, 7]. Conversely, before immune‐checkpoint inhibitors (ICIs) became widely adopted, dMMR/MSI‐H status in metastatic (Stage IV) or recurrent CRC was associated with even worse outcomes compared to pMMR/MSS CRCs [8, 9, 10]. In Stage III disease following curative resection, however, the prognostic significance of dMMR/MSI‐H status remains controversial. While some studies suggest a trend towards a better prognosis, a consistent and statistically robust survival advantage over pMMR/MSS has not been demonstrated, with some meta‐analyses showing no significant survival benefit in Stage III [4, 5, 11, 12, 13]. This complex prognostic landscape, characterized by stage‐dependent patterns in prevalence and outcome, indicates that dMMR/MSI‐H CRC comprises biologically heterogeneous subsets. Although the BRAF V600E mutation is a well‐established adverse prognostic factor in CRC, its negative prognostic impact appears confined to pMMR/MSS CRCs across all stages, including Stage III disease [4, 14, 15, 16]. Therefore, clarifying the heterogeneous subgroups within dMMR/MSI‐H CRC is essential for accurate risk stratification and the development of more personalized therapeutic strategies, especially in postoperative Stage III disease, where the prognostic value of dMMR/MSI‐H status remains uncertain.

CRC exhibits various histological subtypes. While well‐ and moderately differentiated (Wel/Mod) adenocarcinomas constitute the majority of cases, Por and Muc adenocarcinomas are typically associated with aggressive clinical behavior and poorer survival outcomes in unselected cohorts, which primarily represent pMMR/MSS tumors [17, 18, 19]. Paradoxically, dMMR/MSI‐H CRCs, which generally correlate with a favorable prognosis in non metastatic settings, are disproportionately enriched for both Por and Muc histological types. Some studies have investigated the prognostic association of Por CRCs in conjunction with MMR/MSI status, showing no statistically significant results, possibly due to small sample size or the lack of stage‐stratified analyses [18, 20]. Moreover, MMR/MSI status was not an independent predictor of survival in large cohorts of Muc CRC [3, 21]. Given the complex and sometimes conflicting evidence regarding the stage‐dependent prognostic impact of dMMR/MSI‐H CRC in association with Por and Muc histological subtypes, the precise prognostic implications of these histological features within the dMMR/MSI‐H CRC subset remain largely undetermined.

In the present study, we conducted a single‐center, retrospective, exploratory study of patients with CRC across various stages (0–IV) to examine clinicopathological and prognostic characteristics between dMMR/MSI‐H and pMMR/MSS CRCs. We further aimed to explore potential prognostic associations of histological types specifically in Stage III disease, and more specifically within Stage III dMMR/MSI‐H CRCs.

## 2 Methods

### 2.1 Study Population

We enrolled patients with histologically confirmed Stages 0–IV colorectal adenocarcinoma who underwent surgical resection of the primary tumor between 2004 and 2024 at Fukushima Medical University Hospital. Tumors were classified according to the Japanese Classification of Colorectal, Appendiceal, and Anal Carcinoma (JCCRC) [22]. Patients who received preoperative chemotherapy or radiotherapy were excluded. MSI information was available for 289 patients from medical records. MSI testing has been performed routinely for virtually all patients undergoing resection of primary CRC at our institution since late 2021, whereas in earlier years of the study period it was generally performed at the time of recurrence or when clinically indicated. MMR status by immunohistochemistry (IHC) was available for 573 patients, as determined in our previous studies [23, 24, 25, 26]. In addition, we obtained formalin‐fixed paraffin‐embedded (FFPE) whole tissue sections from 326 patients to newly determine MMR status by IHC for the present study. Collectively, MMR/MSI status was ascertained by MSI testing and/or MMR‐IHC in 1127 Stages 0–IV CRCs, which were included in the final analysis. Clinical and pathological information was obtained retrospectively from medical records. For survival analyses, 900 patients with Stages I–III CRC who underwent curative (R0) resection were included in the analyses of relapse‐free survival (RFS) and overall survival (OS). RFS was chosen as the main outcome of interest to provide a less treatment‐confounded assessment of baseline prognostic associations, whereas OS was examined as a secondary, exploratory outcome and presented as complementary information. Because OS is substantially influenced by heterogeneous post‐recurrence management, including cytotoxic or targeted therapies, metastasectomy, and ICIs for dMMR/MSI‐H CRC, its interpretability with respect to baseline prognostic factors is limited. RFS was defined as the time from the date of surgery to the date of first relapse. OS was defined as the time from surgery to death from any cause. The median follow‐up period was 58.7 months.

### 2.2 Determination of MMR/MSI Status

IHC for MMR proteins (MLH1, MSH2, MSH6, and PMS2; MMR‐IHC) was performed as previously described [24]. Briefly, 4‐μm‐thick sections were deparaffinized and rehydrated. Endogenous peroxidase activity was blocked, antigens were retrieved, and slides were incubated overnight at 4°C with primary antibodies, including MLH1 (mouse; clone ES05; M3640; Dako/Agilent Technologies; 1:50), PMS2 (rabbit; clone EP51; M3647; Dako/Agilent Technologies; 1:50), MSH2 (mouse; clone FE11; M3639; Dako/Agilent Technologies; 1:50), and MSH6 (rabbit; clone EP49; M3646; Dako/Agilent Technologies; 1:400). Sections were incubated with horseradish peroxidase (HRP)‐conjugated anti‐rabbit or anti‐mouse secondary antibodies (K4001 or K4003; Dako/Agilent Technologies), visualized with diaminobenzidine (DAB), and nuclei were counterstained. Tumors exhibiting loss of at least one MMR protein were defined as dMMR, and tumors with intact MMR protein expression were classified as pMMR. MSI testing data were obtained from medical records, with MSI‐high or MSI‐positive classified as MSI‐H, and MSI‐low, MSI‐negative, or microsatellite‐stable classified as MSS.

### 2.3 Statistical Analysis

Fisher's exact test, χ^2^ test, or unpaired t‐test with Welch's correction was used to determine differences between two variables, where appropriate. Cumulative survival was estimated by the Kaplan–Meier method, and differences between two groups were analyzed by the log‐rank test. Cox proportional hazard regression was used to compute univariable and multivariable hazard ratios (HR) and 95% confidence intervals (CI). Statistical analyses were performed using Graph Pad Prism 9 (Graph Pad Software, San Diego, CA). A value of *p* < 0.05 was considered to be significant.

## 3 Results

### 3.1 Characteristics of dMMR/MSI‐H and pMMR/MSS CRC

**FIGURE 1:**
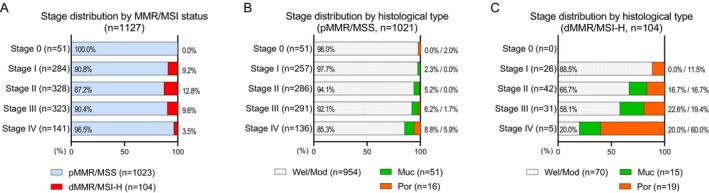
Stage distribution of MMR/MSI phenotypes and histological subtypes in colorectal cancer. (A) Proportions (%) of pMMR/MSS and dMMR/MSI‐H tumors by pathological stage. (B, C) Proportions (%) of histological subtypes by pathological stage in pMMR/MSS (B) and dMMR/MSI‐H (C), showing well/moderately differentiated (Wel/Mod), mucinous (Muc), and poorly differentiated (Por) adenocarcinomas. dMMR/MSI‐H, mismatch repair‐deficient/microsatellite instability–high; pMMR/MSS, mismatch repair‐proficient/microsatellite stable.

In this study, MMR/MSI status was determined by MSI testing in 289 tumors and/or by MMR‐IHC in 902 tumors. Among the 64 tumors analyzed by both MMR/MSI assays, concordance was 98.4%. Of 1127 CRCs included in the analysis, 104 tumors (9.2%) were identified as dMMR/MSI‐H, while 1023 tumors (90.8%) exhibited pMMR/MSS (Table 1). Regarding clinicopathological features, dMMR/MSI‐H tumors were significantly more frequent among females (59.6%, *p* < 0.0001) and were predominantly located in the right colon (81.7%, *p* < 0.0001). We observed no significant differences between dMMR/MSI‐H and pMMR/MSS CRCs in age at diagnosis, T‐stage, lymphatic invasion, venous invasion, or N‐stage (*p* > 0.05). Regarding histological types, the vast majority of pMMR/MSS tumors were Wel/Mod histology (93.3%), with a low proportion of Por (1.6%) and Muc adenocarcinomas (5.0%). In contrast, dMMR/MSI‐H tumors were significantly more often Por (18.3%) or Muc (14.4%) histology (*p* < 0.0001). The stage distribution also differed significantly by MMR/MSI status (*p* = 0.0035), showing that dMMR/MSI‐H was less frequent in Stage IV CRC (4.8%) and was not found in Stage 0 CRC (0.0%). When focusing on the prevalence within each stage, dMMR/MSI‐H was most frequently found in Stage II CRCs (12.8%), followed by Stage III (9.6%), Stage I (9.2%), and Stage IV CRCs (3.5%) (Figure 1A).

**TABLE 1:** Clinicopathological characteristics of 1127 patients with Stage 0–IV colorectal cancer according to MMR/MSI status.

	Total	pMMR/MSS	dMMR/MSI‐H	*p*
	*n* = 1127	*n* = 1023 (90.8%)	*n* = 104 (9.2%)	
Age				
Mean ± SD	68.72 ± 11.75	68.54 ± 11.60	70.49 ± 13.09	0.1461
Sex				
Male	671 (59.5%)	629 (61.5%)	42 (40.4%)	**< 0.0001**
Female	456 (40.5%)	394 (38.5%)	62 (59.6%)	
Location				
Right	433 (38.4%)	348 (34.0%)	85 (81.7%)	**< 0.0001**
Left	694 (61.6%)	675 (66.0%)	19 (18.3%)	
Histological type				
Well‐moderately differentiated	1024 (90.9%)	954 (93.3%)	70 (67.3%)	**< 0.0001**
Poorly differentiated	35 (3.1%)	16 (1.6%)	19 (18.3%)	
Mucinous	66 (5.9%)	51 (5.0%)	15 (14.4%)	
Signet‐ring cell/Undifferentiated	2 (0.2%)	2 (0.2%)	0 (0.0%)	
T‐stage				
Tis	52 (4.6%)	52 (5.1%)	0 (0.0%)	0.1903
T1	165 (14.6%)	150 (14.7%)	15 (14.4%)	
T2	181 (16.1%)	166 (16.2%)	15 (14.4%)	
T3	417 (37.0%)	376 (36.8%)	41 (39.4%)	
T4	311 (27.6%)	279 (27.3%)	32 (30.8%)	
TX	1 (0.1%)	0 (0.0%)	1 (1.0%)	
Lymphatic invasion				
Absent	508 (45.1%)	454 (44.4%)	54 (51.9%)	0.1488
Present	618 (54.8%)	568 (55.5%)	50 (48.1%)	
Not available	1 (0.1%)	1 (0.1%)	0 (0.0%)	
Venous invasion				
Absent	283 (25.1%)	249 (24.3%)	34 (32.7%)	0.0745
Present	843 (74.8%)	773 (75.6%)	70 (67.3%)	
Not available	1 (0.1%)	1 (0.1%)	0 (0.0%)	
N‐stage				
N0	696 (61.8%)	627 (61.3%)	69 (66.3%)	0.4151
N1	316 (28.0%)	289 (28.3%)	27 (26.0%)	
N2–N3	112 (9.9%)	105 (10.3%)	7 (6.7%)	
NX	3 (0.3%)	2 (0.2%)	1 (1.0%)	
M‐stage				
M0	986 (87.5%)	887 (86.7%)	99 (95.2%)	**0.0127**
M1	141 (12.5%)	136 (13.3%)	5 (4.8%)	
Stage				
Stage 0	51 (4.5%)	51 (5.0%)	0 (0.0%)	**0.0035**
Stage I	284 (25.2%)	258 (25.2%)	26 (25.0%)	
Stage II	328 (29.1%)	286 (28.0%)	42 (40.4%)	
Stage III	323 (28.7%)	292 (28.5%)	31 (29.8%)	
Stage IV	141 (12.5%)	136 (13.3%)	5 (4.8%)	

*Note:* Bold values denote statistical significance at the *p* < 0.05 level.

Abbreviations: dMMR/MSI‐H, mismatch repair‐deficient/microsatellite instability–high; pMMR/MSS, mismatch repair‐proficient/microsatellite stable; SD, standard deviation.

### 3.2 Stage‐Dependent Histological Shift Within the dMMR/MSI‐H CRC

We aimed to determine the implications of MMR/MSI status in the context of disease stage and associated histological phenotypes. As depicted in Figure 1B, within the pMMR/MSS CRC cohort, Wel/Mod histology consistently predominated across Stages 0–III (92.1%–98.0%), with Por (0.0%–2.0%) and Muc (0.0%–6.2%) subtypes remaining notably infrequent. In Stage IV pMMR/MSS CRCs, the proportion of Por and Muc histological types increased to 5.9% and 8.8%, respectively. Conversely, in dMMR/MSI‐H CRCs, a notable stage‐dependent redistribution of histological subtypes was evident. In Stage I, nearly 90% of tumors were classified as Wel/Mod, with Por adenocarcinomas accounting for a relatively small fraction (11.5%) (Figure 1C). However, the proportion of Por histology increased progressively from 16.7% in Stage II and 19.4% in Stage III to 60.0% in Stage IV. Concurrently, Muc adenocarcinomas represented a consistent proportion, ranging from 16.7% to 22.6%, across Stages II–IV dMMR/MSI‐H CRCs.

### 3.3 Prognostic Values of MMR/MSI Status and Histological Types in Stage I–III CRC

**FIGURE 2:**
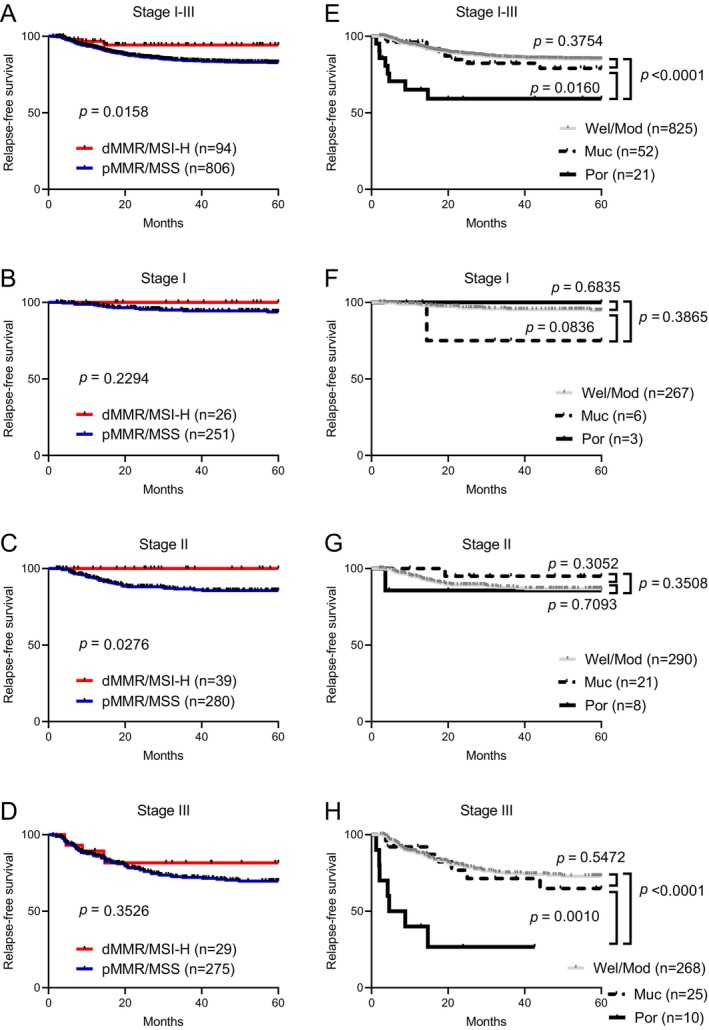
Relapse‐free survival by MMR/MSI status and histological subtype in Stages I–III colorectal cancer. (A–D) Kaplan–Meier curves comparing dMMR/MSI‐H versus pMMR/MSS in (A) Stages I–III combined and, separately, (B) Stage I, (C) Stage II, and (D) Stage III. (E‐H) Kaplan–Meier curves by histological subtype—well/moderately differentiated (Wel/Mod), mucinous (Muc), and poorly differentiated (Por) adenocarcinomas—in (E) Stages I–III combined and, separately, (F) Stage I, (G) Stage II, and (H) stage III. dMMR/MSI‐H, mismatch repair‐deficient/microsatellite instability–high; pMMR/MSS, mismatch repair‐proficient/microsatellite stable.

We investigated the association of MMR/MSI status with RFS in a total of 900 patients with curatively resected Stage I–III CRCs (Figure 2A–H). This analysis included 96 dMMR/MSI‐H and 804 pMMR/MSS CRC patients, and showed that dMMR/MSI‐H was associated with better RFS in Stages I–III (Figure A, *p* = 0.0158). Strikingly, none of the Stage I or II patients with dMMR/MSI‐H CRCs experienced recurrence after surgery (Figure 2B,C). Their favorable prognostic impact on RFS, compared with pMMR/MSS CRCs, did not achieve statistical significance in Stage I (Figure 2B, *p* = 0.2294), but was statistically significant in Stage II (Figure 2C, *p* = 0.0276). Conversely, no significant RFS difference was observed between dMMR/MSI‐H and pMMR/MSS in Stage III disease (Figure 2D, *p* = 0.3526). Histological types also showed stage‐dependent survival differences (Figure 2E–H). In Stage I–III CRC, Por histology was significantly associated with poorer RFS compared with Wel/Mod and Muc histological types (Figure 2E, *p* < 0.0001 and *p* = 0.0160, respectively). Although no significant RFS difference was observed in Stages I and II (Figure 2F,G), patients with Por CRC had significantly worse RFS compared with Wel/Mod and Muc adenocarcinomas in Stage III disease (Figure 2H, *p* < 0.0001 and *p* = 0.0010, respectively).

OS was also examined using the same stratification, as shown in Figure S1. In Stages I–III combined, OS tended to be better in dMMR/MSI‐H than in pMMR/MSS, but this difference was not statistically significant (Figure S1A, *p* = 0.1001), and no significant OS differences were observed in stage‐specific analyses (Figure S1B–D). By histology, Por showed worse OS than Wel/Mod in Stages I–III combined (Figure S1E, *p* = 0.0137), but this association was not consistently significant when analyzed by individual stage (Figure S1F–H). Overall, the OS patterns broadly paralleled the RFS findings but were statistically less robust.

### 3.4 Independent Prognostic Significance of Poorly Differentiated Adenocarcinomas in Stage III CRC

We then specifically investigated Stage III disease, for which the prognostic significance of MMR/MSI status remains equivocal in both our current study (Figure 2D) and previous reports [4, 5, 11, 12, 13]. To examine potential confounding by adjuvant chemotherapy, Stage III cases were stratified by regimen into three groups: surgery alone, fluoropyrimidine (FP) monotherapy, and FP plus oxaliplatin combination (FP + OX). Kaplan–Meier analyses showed no statistically significant differences in RFS by adjuvant regimen in all Stage III patients (*p* = 0.3632), in the dMMR/MSI‐H subgroup (*p* = 0.7935), or in the pMMR/MSS subgroup (*p* = 0.2329) (Figure S2A–C).

Cox proportional hazards models for RFS were applied to 304 patients with Stage III CRC. Multivariable models were adjusted for age, sex, tumor location, histological type, T‐stage (T1–3 vs. T4), N‐stage (N1 vs. N2–3), lymphatic invasion, venous invasion, adjuvant chemotherapy (surgery alone vs. FP, or FP + OX), and MMR/MSI status. As shown in Table 2, univariable analyses identified Por histology (HR: 7.07; 95% CI: 2.93–14.54; *p* < 0.0001), higher T‐stage (HR: 1.67; 95% CI: 1.07–2.62; *p* = 0.024), higher N‐stage (HR: 2.54; 95% CI: 1.54–4.07; *p* = 0.0002), and the presence of venous invasion (HR: 3.32; 95% CI 1.24–13.54; *p* = 0.042) as significantly associated with poorer RFS. In multivariable analyses, Por histology (HR 14.63; 95% CI: 5.41–34.38; *p* < 0.0001), higher T‐stage (HR: 1.67; 95% CI: 1.04–2.69; *p* = 0.033), and higher N‐stage (HR: 3.05; 95% CI: 1.82–4.98; *p* < 0.0001) remained independently associated with worse RFS. Muc histology also emerged as a significant prognostic factor in multivariable analyses (HR: 2.57; 95% CI: 1.03–5.51; *p* = 0.025).

**TABLE 2:** Univariable and multivariable Cox proportional hazard models for relapse‐free survival in stage III colorectal cancer.

		Univariable			Multivariable		
		HR	95% CI	*P*	HR	95% CI	*P*
Age	Continuous	1.02	1.00–1.04	0.092	1.02	0.99–1.05	0.156
Sex	Male vs. Female	0.94	0.60–1.48	0.798	1.11	0.68–1.77	0.678
Tumor location	Right vs. Left	1.22	0.77–2.00	0.408	1.37	0.81–2.38	0.248
Histological type	Wel/Mod vs. Muc	1.27	0.53–2.58	0.552	2.57	1.03–5.51	**0.025**
	Wel/Mod vs. Por	7.07	2.93–14.54	**< 0.0001**	14.63	5.41–35.38	**< 0.0001**
T‐stage	T1–3 vs. T4	1.67	1.07–2.62	**0.024**	1.67	1.04–2.69	**0.033**
N‐stage	N1 vs. N2–3	2.54	1.54–4.07	**< 0.001**	3.05	1.82–4.98	**< 0.0001**
Lymphatic imvasion	Absent vs. Present	1.89	1.04–3.78	0.051	1.32	0.70–2.75	0.417
Venous invasion	Absent vs. Present	3.32	1.24–13.54	**0.042**	3.10	1.06–13.29	0.071
Adjuvant chemotherapy	Surgery alone vs. FP	0.70	0.42–1.17	0.163	0.79	0.45–1.40	0.408
	Surgery alone vs. FP + OX	0.76	0.40–1.40	0.383	0.76	0.36–1.59	0.470
MMR/MSI status	pMMR/MSS vs. dMMR/MSI‐H	0.65	0.23–1.46	0.357	0.35	0.10–0.96	0.060

*Note:* Bold values denote statistical significance at the *p* < 0.05 level.

Abbreviations: CI, confidence interval; dMMR/MSI‐H, mismatch repair‐deficient/microsatellite instability–high; FP + OX, FP plus oxaliplatin combination therapy; FP, fluoropyrimidine monotherapy; HR, hazard ratio; Muc, mucinous adenocarcinoma; pMMR/MSS, mismatch repair‐proficient/microsatellite stable; Por, poorly differentiated adenocarcinoma; Wel/Mod, well/moderately differentiated adenocarcinoma.

### 3.5 Stratified Analyses of Stage III CRC by MMR/MSI Status

**FIGURE 3:**
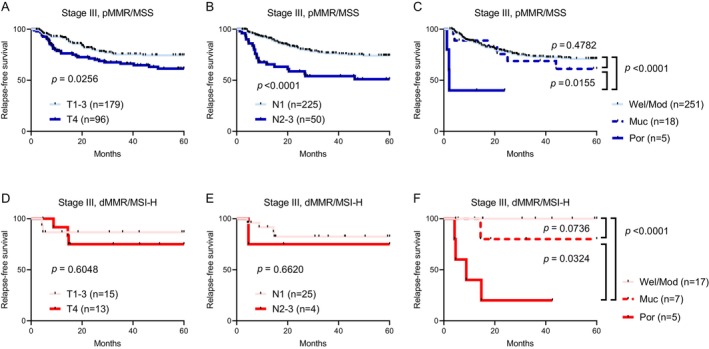
Relapse‐free survival in Stage III colorectal cancer stratified by MMR/MSI status. (A–C) pMMR/MSS cohort. Kaplan–Meier curves according to (A) T‐stage, (B) N‐stage, and (C) histological subtype—well/moderately differentiated (Wel/Mod), mucinous (Muc), poorly differentiated (Por). (D–F) dMMR/MSI‐H cohort. Kaplan–Meier curves according to (D) T‐stage, (E) N‐stage, and (F) histological subtype. dMMR/MSI‐H, mismatch repair‐deficient/microsatellite instability–high; pMMR/MSS, mismatch repair‐proficient/microsatellite stable.

Beyond T‐stage and N‐stage, histological types also correlated with RFS in Stage III disease, independent of MMR/MSI status (Table 2). This observation is consistent with our finding of a stepwise enrichment of Por histology during tumor progression in dMMR/MSI‐H CRCs (Figure 1C). We therefore hypothesized that Por histology represents an aggressive phenotype particularly in dMMR/MSI‐H CRCs and that it could further classify dMMR/MSI‐H CRCs into prognostic subgroups. As shown in Figure 3A–F, Stage III CRCs were subsequently stratified into pMMR/MSS (Figure 3A–C) and dMMR/MSI‐H (Figure 3D–F), and RFS analyses were conducted according to T‐stage (Figure 3A,D), N‐stage (Figure 3B,E) and histological type (Figure 3C,F). In Stage III pMMR/MSS CRCs, we observed a significant RFS difference between T1–3 and T4 (Figure 3A, *p* = 0.0256), as well as between N1 and N2–3 tumors (Figure 3B, *p* < 0.0001). However, in contrast to pMMR/MSS, neither T‐stage nor N‐stage was associated with RFS in Stage III dMMR/MSI‐H CRCs (Figure 3D,E). Notably, in both pMMR/MSS and dMMR/MSI‐H CRCs, Por adenocarcinomas were significantly associated with poorer RFS compared with Wel/Mod (Figure 3C,F, *p* < 0.0001 and *p* = 0.0155, respectively) or Muc subtypes (Figure 3C,F, *p* < 0.0001 and *p* = 0.0324, respectively). Most strikingly, none of the 17 Stage III patients with Wel/Mod dMMR/MSI‐H CRC experienced recurrence (0.0%), whereas 4 of 5 Stage III patients with Por dMMR/MSI‐H CRC had early recurrence (80.0%) within 15 months after surgery (Figure 3F).

## 4 Discussion

This was a single‐center retrospective study analyzing 1127 patients with Stage 0–IV CRC. We investigated the intricate relationship between MMR/MSI status, histological types, and disease stage in CRC, aiming to refine our understanding of their prognostic relevance. Our findings suggest distinct histological shifts during tumor progression and provide additional insight into stage‐dependent prognostic implications, particularly highlighting the role of Por histology within Stage III dMMR/MSI‐H tumors.

Our study confirmed many established clinicopathological and prognostic associations of dMMR/MSI‐H CRC. MMR/MSI status was determined for all tumors included in this study, and a subset was analyzed by both MMR‐IHC and PCR‐based MSI testing, demonstrating a 98.4% concordance, consistent with previously reported high concordance between these methods [27, 28]. This supports the robustness of MMR/MSI status determination, with both methods widely adopted in current clinical practice. We identified 104 patients with dMMR/MSI‐H CRC (9.2%), more often at Stage II (12.8%) and rarely at Stage IV disease (3.5%). These tumors were associated with female sex, right‐sided colon location, and Por or Muc histology. These general characteristics and the prevalence of dMMR/MSI‐H tumors in our cohort are broadly consistent with existing epidemiological and clinicopathological data [2, 5, 8]. Additionally, all 51 Stage 0 tumors were pMMR/MSS in our cohort, which is consistent with a previous report analyzing 41 Stage 0 tumors [29]. In RFS analyses of 900 patients with Stages I–III CRC who underwent curative resection, dMMR/MSI‐H tumors showed a favorable prognostic trend; notably, no patients with dMMR/MSI‐H CRC experienced postoperative recurrence in Stages I and II. The survival advantage of dMMR/MSI‐H reached statistical significance in Stage II but not in Stage I. In Stage III disease, RFS did not differ by MMR/MSI status. This stage‐dependent prognostic pattern, benefit in Stage II and an inconclusive effect in Stage III, aligns with numerous previous studies [4, 5, 6, 7, 11, 12, 13].

A key focus of our investigation was the interplay between histology and disease stage, particularly as it relates to MMR/MSI status. Our findings revealed notable histological patterns. In pMMR/MSS CRCs, Wel/Mod histology consistently dominated across Stages 0–III (92.1%–98.0%), while the Por subtype remained rare (0.0%–2.0%). In Stage IV pMMR/MSS CRCs, the proportions of Por adenocarcinomas increased to 5.9%. Conversely, in dMMR/MSI‐H CRC, a striking stage‐dependent histological shift was evident, marked by a progressive expansion of Por histology from 11.5% in Stage I to 60.0% in Stage IV. Indeed, dMMR/MSI‐H CRCs often exhibit Por histology yet paradoxically tend to have a favorable prognosis in earlier stages. Therefore, the enrichment of the more aggressive Por phenotype in later stages, particularly in dMMR/MSI‐H CRCs, suggests that histological subtype alone is not a reliable prognostic marker in CRC without considering disease stage and MMR/MSI status. We further performed detailed RFS analyses specifically for Stage III, where MMR/MSI status alone proved inconclusive. Our Cox analyses in Stage III CRCs revealed that Por histology, higher T‐stage, and higher N‐stage were significant independent prognostic factors for poorer RFS. This underscores the importance of traditional clinicopathological features in risk stratification for Stage III CRC. We then conducted stratified analyses of Stage III CRC by MMR/MSI status, which yielded additional observations. Despite overall correlations of T‐stage, N‐stage, and histological type with RFS in Stage III independent of MMR/MSI status, we observed a divergence between pMMR/MSS and dMMR/MSI‐H. While significant RFS differences were observed between T1–T3 and T4, and between N1 and N2–3, in Stage III pMMR/MSS CRCs, neither T‐stage nor N‐stage was associated with RFS in Stage III dMMR/MSI‐H CRCs, likely owing to small sample size and limited statistical power. Notably, in both pMMR/MSS and dMMR/MSI‐H CRCs, Por adenocarcinomas had significantly poorer RFS compared with Wel/Mod or Muc subtypes. Strikingly, none of the patients with Wel/Mod dMMR/MSI‐H CRC recured after curative resection, whereas 80.0% of Stage III patients with Por dMMR/MSI‐H CRC experienced early recurrence within 15 months of surgery. Therefore, despite the overall favorable prognosis of dMMR/MSI‐H tumors, Por histology, as an aggressive phenotype, may be associated with worse outcomes, attenuating their survival advantage. This also reinforces the potential intertumor heterogeneity among dMMR/MSI‐H CRCs, and histological types may help further classify these tumors into prognostic subgroups, especially in Stage III. From a clinical perspective, our data suggest that Por histology in Stage III dMMR/MSI‐H CRC may help identify a subgroup at increased risk of early recurrence. This information may be useful for postoperative risk stratification and for considering more intensive adjuvant chemotherapy and/or tailored surveillance strategies, although such approaches should be validated in larger, prospective studies.

Mechanistically, the stage‐dependent enrichment of Por histology in dMMR/MSI‐H CRC may reflect the progressive accumulation of genetic alterations in genes that regulate cellular differentiation. For example, oncogenic BRAF mutations and defects in differentiation‐maintaining pathways, such as the TGFβ and Wnt/β‐catenin signaling axes, could promote dedifferentiation during tumor progression. Furthermore, this histological pattern may be shaped by immune‐mediated selective pressure, a process consistent with cancer immunoediting. In this context, highly immunogenic, more differentiated tumor cells may be recognized and eliminated by the host immune system, particularly in early stages. In contrast, immune‐evasive clones, potentially characterized by aggressive, poorly differentiated phenotypes, may be preferentially selected at advanced stages. While these mechanistic links remain speculative and were not directly assessed in this study, they warrant validation in future molecular and immunologic investigations.

Our study has several limitations. Our study has several limitations. First, this was a single‐center, retrospective analysis from a Japanese tertiary referral hospital and is therefore subject to inherent selection bias, missing or incomplete clinical information, and residual confounding, despite multivariable adjustment. Because the study included only patients who underwent surgical resection of the primary tumor without preoperative treatment, the cohort does not capture patients with unresectable or medically inoperable disease, those managed non‐operatively, or those with very poor performance status or severe comorbidities. As a result, our study population mainly represents relatively fit patients selected for surgical treatment of the primary lesion, and the survival analyses were further restricted to Stages I–III cases who underwent curative (R0) resection. Patterns of referral, surgical management, adjuvant therapy, and follow‐up in our institution may not be fully generalizable to other settings or populations. Second, although our cohort was large, the numbers in some key molecular–histological subgroups were small, particularly Stage III dMMR/MSI‐H cases with poorly differentiated histology. Effect estimates in these subgroups should therefore be interpreted with caution and regarded as hypothesis‐generating rather than definitive, and external validation in larger cohorts will be required. Third, adjuvant and salvage systemic treatments evolved substantially over the study period. This therapeutic heterogeneity, including the use of oxaliplatin‐containing adjuvant regimens and the more recent introduction of ICIs for recurrent disease, may have influenced outcomes, especially OS. In addition, our primary outcome of interest was RFS, with OS analyzed only as a secondary, exploratory endpoint, as OS is likely to be affected by heterogeneous post‐recurrence therapies and should be interpreted in that context. Fourth, for histological classification we adhered to the JCCRC, which differs from the WHO grading system. In the WHO system, tumor grading is classified by the highest‐grade component, whereas the JCCRC classifies histological subtypes according to the predominant component [30].

In conclusion, this study not only confirms established clinicopathological and prognostic associations of dMMR/MSI‐H CRC and importantly but also provides additional evidence that Por histology, more prevalent in advanced disease, can significantly refine risk stratification within Stage III dMMR/MSI‐H CRC. These findings underscore the potential value of developing refined prognostic models that integrate specific histological features with MMR/MSI status to better inform personalized treatment strategies, particularly in Stage III disease.

## Author Contributions

**Tomoyuki Momma:** conceptualization, supervision, writing – original draft, writing – review and editing, project administration, resources, validation, formal analysis, methodology, investigation. **Hirokazu Okayama:** methodology, conceptualization, data curation, supervision, formal analysis, validation, investigation, funding acquisition, writing – original draft, writing – review and editing, visualization, project administration, resources. **Sohei Hayashishita:** resources, data curation. **Hiroya Suzuki:** data curation, resources. **Masanori Katagata:** data curation, resources. **Takuro Matsumoto:** data curation, resources. **Daisuke Ujiie:** data curation, resources. **Shun Chida:** data curation, resources. **Wataru Sakamoto:** resources, writing – original draft, conceptualization, supervision. **Koji Kono:** conceptualization, writing – original draft, writing – review and editing, project administration, supervision.

## Funding

This work was supported by grants from Japan Society for the Promotion of Science (JSPS) KAKENHI Grant Number 25 K12010.

## Ethics Statement

The study was conducted in accordance with the Declaration of Helsinki and was approved by the Institutional Review Board of Fukushima Medical University (REC2024‐041).

## Conflicts of Interest

The authors declare no conflicts of interest.

## Supporting information


**Figure S1:** Overall survival by MMR/MSI status and histological subtype in Stages I–III colorectal cancer. (A–D) Kaplan–Meier curves comparing dMMR/MSI‐H versus pMMR/MSS in (A) Stages I–III combined and, separately, (B) Stage I, (C) Stage II, and (D) Stage III. (E–H) Kaplan–Meier curves by histological subtype, well/moderately differentiated (Wel/Mod), mucinous (Muc), and poorly differentiated (Por), in (E) Stages I–III combined and, separately, (F) Stage I, (G) Stage II, and (H) Stage III.

**Figure S2:** Relapse‐free survival in Stage III colorectal cancer according to adjuvant chemotherapy, stratified by MMR/MSI status. (A–C) Kaplan–Meier curves comparing surgery alone, adjuvant fluoropyrimidine monotherapy (FP), and adjuvant FP plus oxaliplatin combination therapy (FP + OX), in all Stage III (A), dMMR/MSI‐H (B), and pMMR/MSS (C).
