# Supplementary figures and images for: Prognostic Impact of Histologic Subtypes in Mismatch Repair‐Deficient/Microsatellite Instability‐High Colorectal Cancer: A Single‐Center Retrospective Study of 1127 Stage 0–IV Patients
Source: Ann Gastroenterol Surg. 2025 Dec 30;10(3):760–9. doi: 10.1002/ags3.70159 (PMC13178275; doi:10.1002/ags3.70159)

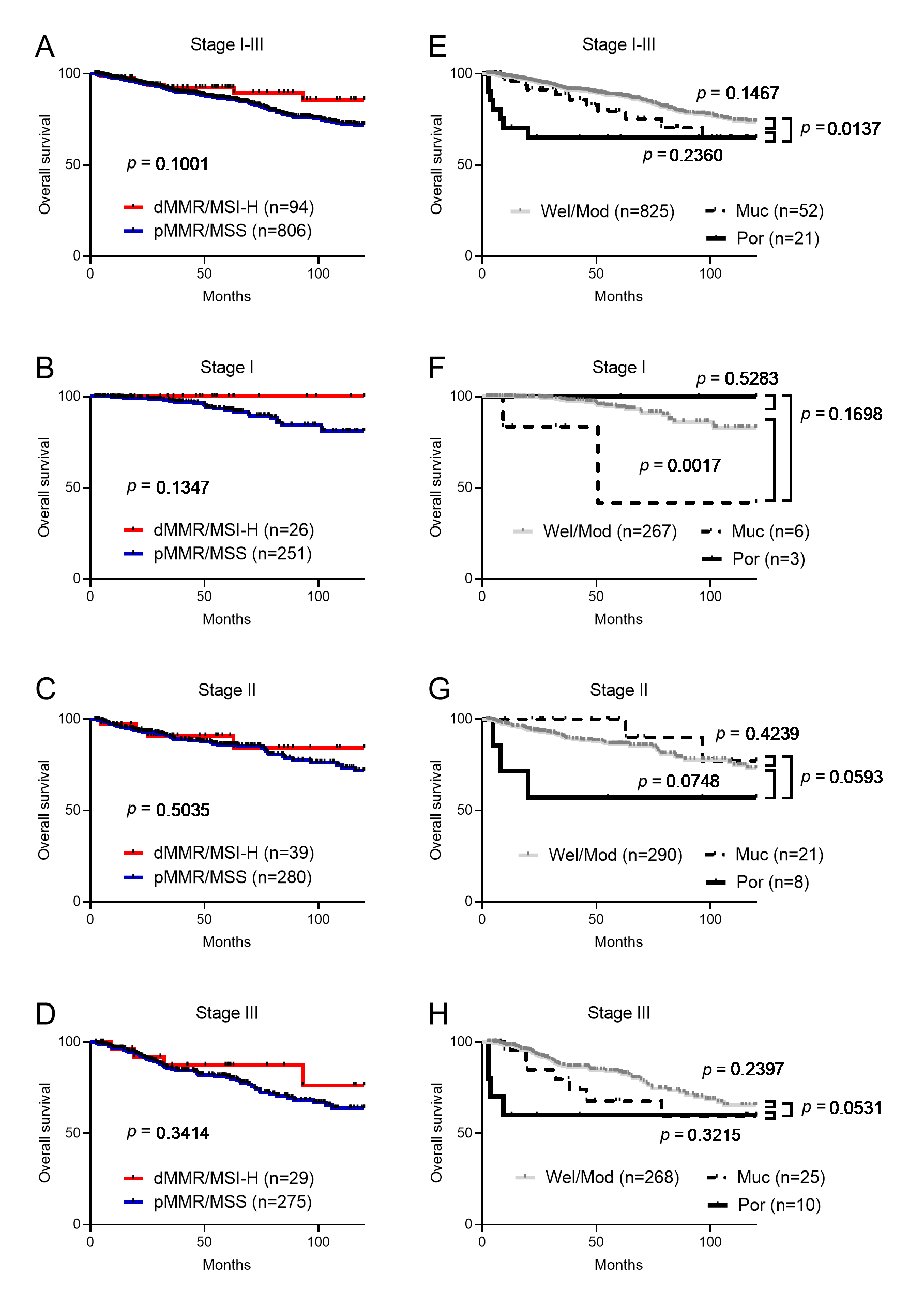

Supplement: Supplementary file 1 — Figure S1: Overall survival by MMR/MSI status and histological subtype in Stages I–III colorectal cancer. (A–D) Kaplan–Meier curves comparing dMMR/MSI‐H versus pMMR/MSS in (A) Stages I–III combined and, separately, (B) Stage I, (C) Stage II, and (D) Stage III. (E–H) Kaplan–Meier curves by histological subtype, well/moderately differentiated (Wel/Mod), mucinous (Muc), and poorly differentiated (Por), in (E) Stages I–III combined and, separately, (F) Stage I, (G) Stage II, and (H) Stage III. [file AGS3-10-760-s001.tif]

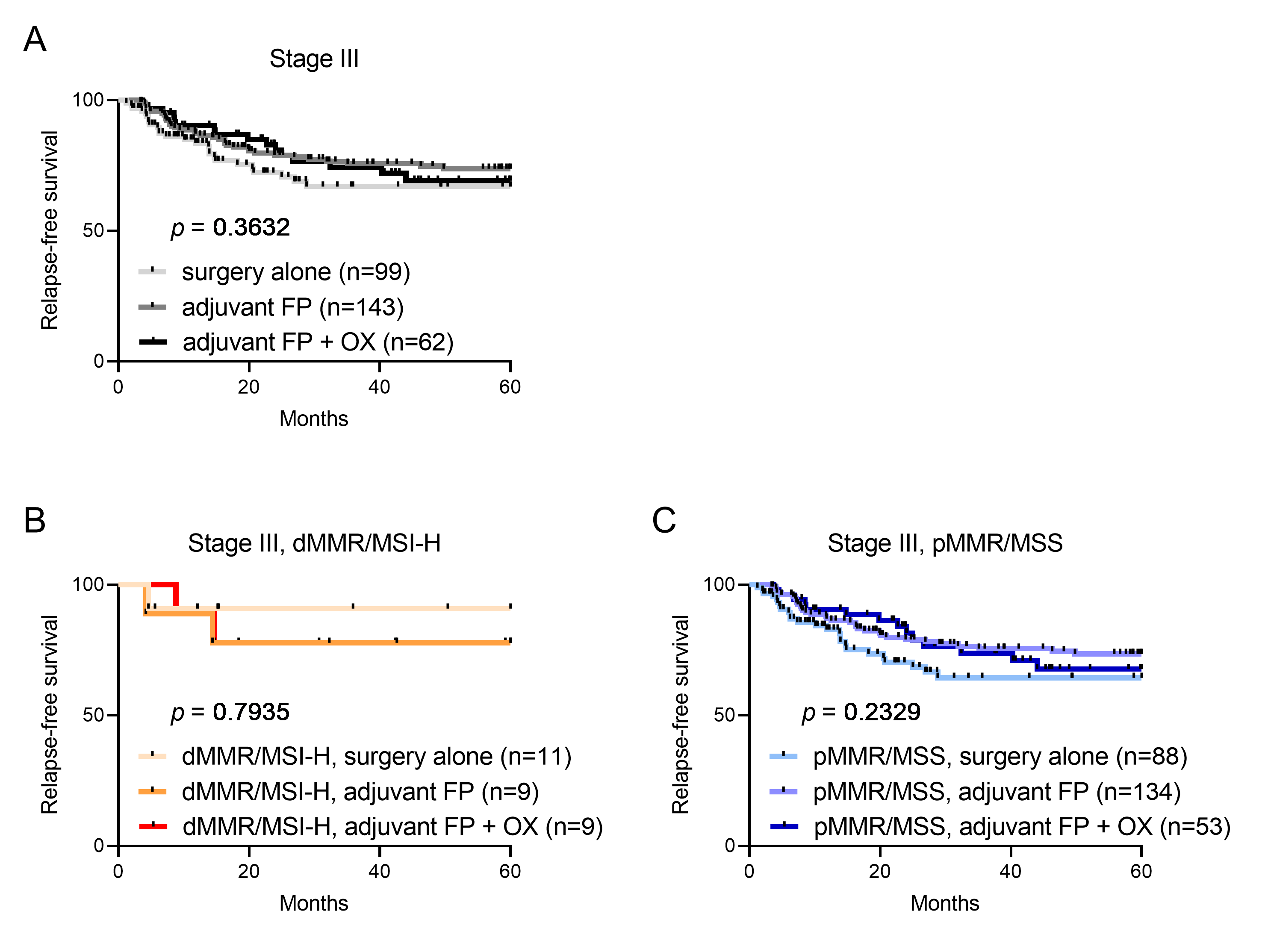

Supplement: Supplementary file 2 — Figure S2: Relapse‐free survival in Stage III colorectal cancer according to adjuvant chemotherapy, stratified by MMR/MSI status. (A–C) Kaplan–Meier curves comparing surgery alone, adjuvant fluoropyrimidine monotherapy (FP), and adjuvant FP plus oxaliplatin combination therapy (FP + OX), in all Stage III (A), dMMR/MSI‐H (B), and pMMR/MSS (C). [file AGS3-10-760-s002.tif]
